# Prospects of Directly Reprogrammed Adult Human Neurons for Neurodegenerative Disease Modeling and Drug Discovery: iN vs. iPSCs Models

**DOI:** 10.3389/fnins.2020.546484

**Published:** 2020-11-19

**Authors:** Ying Zhang, Xinyang Xie, Jiangnan Hu, Kazi Sabrina Afreen, Chun-Li Zhang, Qichuan Zhuge, Jianjing Yang

**Affiliations:** ^1^Zhejiang Provincial Key Laboratory of Aging and Neurological Disorder Research, The First Affiliated Hospital of Wenzhou Medical University, Wenzhou, China; ^2^Department of Neurosurgery, The First Affiliated Hospital of Wenzhou Medical University, Wenzhou, China; ^3^International Department of The Affiliated High School of South China Normal University (HFI), Guangzhou, China; ^4^Department of Pharmaceutical Sciences, University of North Texas Health Science Center, Fort Worth, TX, United States; ^5^Department of Microbiology & Immunology, Rosalind Franklin University of Medicine and Science, North Chicago, IL, United States; ^6^Department of Molecular Biology, UT Southwestern Medical Center, Dallas, TX, United States

**Keywords:** iN, iPSCs, disease modeling, drug screening, neurodegenerative disease

## Abstract

A reliable disease model is critical to the study of specific disease mechanisms as well as for the discovery and development of new drugs. Despite providing crucial insights into the mechanisms of neurodegenerative diseases, translation of this information to develop therapeutics in clinical trials have been unsuccessful. Reprogramming technology to convert adult somatic cells to induced Pluripotent Stem Cells (iPSCs) or directly reprogramming adult somatic cells to induced Neurons (iN), has allowed for the creation of better models to understand the molecular mechanisms and design of new drugs. In recent times, iPSC technology has been commonly used for modeling neurodegenerative diseases and drug discovery. However, several technological challenges have limited the application of iN. As evidence suggests, iN for the modeling of neurodegenerative disorders is advantageous compared to those derived from iPSCs. In this review, we will compare iPSCs and iN models for neurodegenerative diseases and their potential applications in the future.

## Introduction

Neurodegenerative diseases comprised of a group of complicated disorders of the central nervous system among the aged population. To design effective treatment strategies to cure these diseases, scientists are in desperate need of convenient and reliable disease models. Previous neurodegenerative disease models based on genetic manipulations include transgene integration or gene knockout systems. These systems can only be utilized partially to understand disease mechanisms, pathology, and progression (Hargus et al., 2014; Heilker et al., 2014; Imaizumi and Okano, 2014; Zhao et al., 2014). These current models cannot be used as accurate models for neurodegenerative diseases especially due to specific limitations. First, although the fibroblasts or disease-associated mutation transformed cell lines of patients have enabled detailed mechanistic studies to be carried out, the biology of cell lines does not resemble the biology of primary neurons (Hargus et al., 2014). Thus it is often unclear whether the mechanisms studied are directly comparable to patients’ pathology. Second, animal models-such as dogs, flies, monkeys, and especially rodents (Zhao et al., 2014), is another method of studying neurodegenerative diseases (Gitler et al., 2017). However, these models often cannot accurately recapitulate human disease and animal models of the sporadic forms of neurodegenerative diseases due to species-specific differences. In addition, it is difficult to manipulate affected cell types in neurodegenerative disorders *in vitro*. Due to these limitations, a number of preclinical trials that aimed to identify drugs have failed to successfully translate into therapeutics in clinical settings (Kraljevic et al., 2004; Ledford, 2011; Ke et al., 2016). In summary, it is important to develop accurate and predictive disease models as they are essential to providing key insights to understanding disease mechanisms and the development of drugs to cure neurodegenerative diseases.

Innovations in cellular reprogramming technology have provided us with a promising tool to solve this problem. Takahashi and Yamanaka (2006) established a unique method of reprogramming somatic cells to iPSCs, which can be differentiated into cell types of all the three germ layers including non-proliferating neurons. The neurons derived from iPSCs would have the same genetic information as the individual patient and can be differentiated from iPSCs. This technology has been utilized by other investigators for neurodegenerative disease modeling (Table 1; Wan et al., 2015; Haston and Finkbeiner, 2016; Liu and Deng, 2016; Csobonyeiova et al., 2017). Moreover, in recent years, the discovery of direct reprogramming technology has enabled the reprogramming of somatic cells to neurons, bypassing the iPSC stage (Vierbuchen et al., 2010; Ambasudhan et al., 2011; Li et al., 2015, 2017; Karow et al., 2018; Tanabe et al., 2018; Xiao et al., 2018). With the advancement of these technologies, scientists have been able to create highly efficient and lineage-specific neurons through the reprogramming of somatic cells (Marro et al., 2011; Xu Z. et al., 2015; Black et al., 2016; Mall et al., 2017). Altogether, these technologies can be used for modeling neurodegenerative diseases (Shi et al., 2017; Sun et al., 2017; Han et al., 2018; Farkhondeh et al., 2019).

**TABLE 1:** Neurons derived from iPSC technology.

**Transcription factors**	**Cell type**	**Techniques**	**Neurons type**	***In vitro*/ *in vivo***	**Efficiency**	**References**
OSKM(Oct4, Sox2, Klf4 and c-Myc)	mouse and human fibroblasts	retroviruses		*in vitro* and *in vivo*	Low and tedious	Takahashi and Yamanaka, 2006; Takahashi et al., 2007
OSNL(Oct4, Sox2,Nanog,LIN28)	human somatic cells	lentivirus		*in vitro*	low	Yu et al., 2007
OSKM with TAV, SB431542, PD0325901 and ascorbic acid	bone marrow– derived mesenchymal cells of marmosets	excisable lentiviral spleen focus-forming virus	Neural progenitors	*in vitro*	high	Wiedemann et al., 2012
OSKM	human adipose- derived stem cells	polycistronic plasmid		*in vitro*		Barbuti et al., 2012
OSKM co-expressing tyrosine hydroxylase	human fibroblasts	an RNA virus (Sendai virus)	dopaminergic neurons	*in vitro* and *in vivo*	significantly surpassed retroviral transduction (0.02%	Fusaki et al., 2009
OSNL	human adipose stromal cells	non-viral minicircle DNA vector		*in vitro*	lower (∼0.005%)	Narsinh et al., 2010
OSKM	human fibroblasts	modified RNA	neuron like cells	*in vitro*	High (2%)	Warren et al., 2010
OSKM	human fibroblasts	OSKM proteins fused with a cell-penetrating peptide	all three embryonic germ layers	*in vitro* and in teratomas	slow and low	Kim et al., 2009
Forskolin, 2-methyl-5-hydroxytryptamine, D4476, VPA, CHIR99021, 616452 and Tranylcypromine	mouse embryonic fibroblasts	small-molecule compounds		*in vitro*	0.2%	Hou et al., 2013
NaB, PD03259, and SB431542	human fibroblasts	upregulates the miR302/367 cluster expression		*in vitro*		Zhang and Wu, 2013

Even though, the mechanisms of iPSCs or iN reprogramming are still unclear (Xu J. et al., 2015; Omole and Fakoya, 2018), there are some obvious differences between iPSCs-derived neurons and iN. Among them, recent studies have indicated that the application of iN for aging-related neurodegenerative diseases would be a better choice, as it does not reset aging information (Mertens et al., 2015, 2018; Tang et al., 2017; Bohnke et al., 2018; Traxler et al., 2019). In this review, we summarize recent studies involving iPSCs and in neurodegenerative disease modeling and its advantages and limitations.

## Reprogramming Somatic Cells to Neuron Cells

### iPSC Technology

In 2006, a phenomenal study conducted in Yamanaka lab demonstrated that viral vectors carrying a combination of pluripotent transcription factors, including Oct4, Sox2, Klf4, and c-Myc (OSKM), were sufficient to effectively reprogram mouse fibroblasts cells to iPSCs (Takahashi and Yamanaka, 2006; Okano and Yamanaka, 2014). In 2007, their laboratory also demonstrated that OSKM could reprogram human fibroblasts to iPSCs by the retroviral system (Takahashi et al., 2007). The generated iPSCs had the potential to be differentiated into all three germ layers of cell type with the unlimited ability of self-renewal. Besides OSKM, the combination of other transcription factors, including Oct4, Sox2, Nanog, and LIN28, has also been demonstrated to be able to convert human somatic cells into iPSCs with a lentiviral system (Yu et al., 2007). In addition, this technology has been successfully used for translating into other somatic cell types, such as neural stem cells (Eminli et al., 2008; Kim et al., 2008), stomach and liver cells (Aoi et al., 2008), mature β lymphocytes (Hanna et al., 2008), melanocytes (Utikal et al., 2009), adipose stem cells (Sun et al., 2009), and keratinocytes (Maherali et al., 2008). iPSC technology provides a platform that can be used as a model system for neurodegenerative diseases to design new therapeutics. However, the current iPSC technology still has some limitations, including low efficiency and a long reprogramming process, which are primarily due to the existence of several roadblocks (Ebrahimi, 2015; Haridhasapavalan et al., 2020). Another problem is that iPSCs may cause cancerous tumor formation due to an undifferentiated pluripotent stem cell after transplantation (Choi and Hong, 2017). In recent years, researchers put tremendous efforts into refining and optimizing approaches to improve reprogramming efficiency and safety (O’Malley et al., 2009; Sommer and Mostoslavsky, 2010, 2013; Gonzalez et al., 2011; Morris and Daley, 2013; Omole and Fakoya, 2018; Borgohain et al., 2019; Haridhasapavalan et al., 2019). Maherali et al. (2008) created a doxycycline-inducible lentiviral system including OSKM, which had a higher frequency of converting primary fibroblasts into iPSCs. This system could even reprogram keratinocytes into iPSCs within 10 days (Maherali et al., 2008). In addition, using lentivirus or retrovirus to deliver OSKM may cause insertional mutagenesis when integrating gene sequences in the genomic DNA of the cells. To improve technical safety, other delivery methods, including non-viral or non-integrating viral vectors, have been attempted, such as protein transduction, the transfection of modified mRNA transcripts, small molecules, sendai virus, and episomal vectors (Sommer and Mostoslavsky, 2010, 2013; Gonzalez et al., 2011; Morris and Daley, 2013; Omole and Fakoya, 2018; Borgohain et al., 2019; Haridhasapavalan et al., 2019). However, compared to the traditional viral gene delivery method, these alternative methods had poorer outcomes.

### iN Technology

After the establishment of iPSC reprogramming technology, researchers are continuously seeking effective ways to improve the reprogramming condition. The main challenge is to rapidly and efficiently change cell fate by reprogramming using minimal transcription factors. In 2010, Vierbuchen and his group succeeded in directly reprogramming mouse fibroblasts to functional neurons by overexpression of three transcription factors, including Ascl1, Brn2, and Myt1l (Vierbuchen et al., 2010). Subsequently, several studies showed some other transcription factors (Ngn2, Ascl1, and Dlx2) also could convert mouse postnatal astrocytes into both GABAergic and cholinergic neurons (Berninger et al., 2007; Heinrich et al., 2011; Xiao et al., 2018; Huang et al., 2019; Wazan et al., 2019). Moreover, only one transcription factor NGN2, when supplemented with chemicals including dorsomorphin and forskolin, could directly reprogram human fibroblasts (MRC5) to neurons (Liu et al., 2013) with high efficiency. The neurons generated are functional and mostly cholinergic neurons (Liu et al., 2013). Only epigenetic chemicals without transcription factors have been demonstrated to directly reprogram human and mouse fibroblasts into functional neuron cells (Hu et al., 2015; Li et al., 2015, 2017; Smith et al., 2016; Qin et al., 2017). Other studies have shown that some defined tissue-specific transcription factors (TFs), such as Sox2, Zfp521 (a single zinc-finger TF), and Ptfa1, directly reprogram human fibroblasts into a neural stem cell (Maucksch et al., 2013; Shahbazi et al., 2016; Xiao et al., 2018). In addition, in Rubio et al. (2016) used the CRISPR/Cas9 platform to inactivate two neurological disorder genes, TSC2 and KCNQ2 and subsequently combined with a multicistronic lentivirus expressing the Ascl1, Lmx1a, and Nurr1 genes to directly convert fibroblasts into neuropathological-resistant neuronal cells. Although several other cell types can also be reprogrammed into neurons, like hepatocytes and pericytes cells (Marro et al., 2011; Karow et al., 2012), fibroblasts are still the most popular original cell type for reprogramming. Together these findings supported that iN can be directly derived from different cell types by certain combinations of transcription factors (Table 2). This technology for the generation of iN from other cell types could be useful for the development of neurological disease models (Ruggieri et al., 2014; Gascon et al., 2017; Gao et al., 2019; Pereira et al., 2019).

**TABLE 2:** Neurons derived from direct reprogramming technology.

**Transcription factors**	**Cell type**	**Techniques**	**Neurons type**	***In vitro*/ *in vivo***	**Efficiency**	**Functional or not**	**References**
ASCL1, NGN2, SOX2, NURR1 and PITX3	human fibroblasts	lentivirus	iN (mostly dopaminergic neurons)	*in vivo*	∼80%	functional electrophysiology	Liu et al., 2011
Ascl1, Brn2, Myt1l	mouse hepatocytes	lentivirus	iN	*in vivo*	>90%	functional electrophysiology	Marro et al., 2011
Sox2 and Mash1	pericyte-derived cells of the adult human cerebral cortex	retrovirus	GABAergic neurons	*in vitro*	∼50%	these iN acquire the ability of repetitive action potential firing and serve as synaptic targets for other neurons	Karow et al., 2012
Brn2, Myt1l, Zic1, Olig2, and Ascl1	Mouse Embryonic fibroblasts	lentivirus	iN (mostly GABAergic and glutamatergic neurons)	*in vitro*	∼50%	functional electrophysiology Synaptic maturation	Vierbuchen et al., 2010
Ascl1, Brn2 and Myt1l	mouse embryonic and postnatal fibroblasts	lentivirus	iN (mostly excitatory neurons)	*in vitro*	19.5%	functional electrophysiology Synaptic maturation	Vierbuchen et al., 2010
Ascl1, Brn2 and Myt1l	mouse and human cells	viral delivery	neurons	*in vivo*	20%	functional	Torper et al., 2013
NeuroD1, Ascl1, Brn2, and Mytl1	human fibroblasts	lentivirus	iN	*in vitro*	∼60%	functional neurons	Pang et al., 2011
Ascl1, Lmx1a, FoxA2, and FEV	human fibroblasts	Dox-inducible lentivirus	serotonergic (i5HT) neurons	*in vitro*	∼25%	exhibited spontaneous electrophysiological activity and had active serotonergic synaptic transmission	Xu Z. et al., 2015
Mash1, Nurr1 and Lmx1a	mouse and human fibroblasts	lentivirus	iN (mostly dopaminergic neurons)	*in vitro*	high	functional electrophysiology	Caiazzo et al., 2011
NGN2 with (Forskolin and dorsomorphin)	human fetal lung fibroblasts	retrovirus	cholinergic neurons	*in vitro*	>90%	characteristic electrophysiological properties	Liu et al., 2013
LDN193189, SB431542, TTNPB, Tzv, CHIR99021, VPA, DAPT, SAG, Purmo	Human astrocytes	with medium	Functional neurons (mainly glutamatergic neurons)	*in vitro*	>90%	functional	Zhang et al., 2015
Forskolin, ISX9, CHIR99021 and SB431542	mouse fibroblasts	with medium	iN	*in vitro*	>90%	functional electrophysiology	Li et al., 2015

## Direct Reprogramming to Generate Specific Neuronal Subtypes

During the early stages, just after the discovery of direct reprogramming technology, investigators paid more attention to the efficacy of reprogramming and whether the neurons generated are physiologically functional. Subsequently, investigators tried to control the reprogramming process to convert somatic cells to specific neuronal subtypes. Reprogramming somatic cells into defined neuronal subtypes is a crucial step for the application of iN reprogramming technology into clinical trials. In recent years, technical improvements in this field have made substantial progress, which would dramatically increase the applications of iN technology.

### Dopaminergic Neurons

Parkinson’s disease is a neurodegenerative disorder with progressive loss of dopaminergic neurons in the midbrain (Alexander, 2004). Thus, using reprogramming technology for the generation of defined dopaminergic neurons could be an interesting approach for the treatment of Parkinson’s disease. According to neuronal system development, several transcription factors play a critical role in the generation and specification of dopaminergic neurons, including Otx2, FoxA1/2 Lmx1a/b, Ascl1, Ngn2, Pitx3, and Nurr1 (Nr4a2) (Arenas et al., 2015). Several studies have reported the successful reprogramming of fibroblasts or astrocytes into induced dopaminergic (iDA) neurons. Among them, the minimal combination is Ascl1, Nurr1, and Lmx1a (Kim et al., 2011; Pfisterer et al., 2011; Torper et al., 2013; Caiazzo et al., 2015). The iDA neurons that are generated are functional, can produce dopamine, and have firing of action potentials and functional D2 auto receptors (Caiazzo et al., 2011). Moreover, transplantation of these functional iDA neurons could improve the behavior deficit caused by the loss of endogenous DA neurons (Dell’Anno et al., 2014). De Gregorio et al. (2018) found that, when combined with transcription factors ASCL1 and NURR1, miR-34b/c could double the yield of transdifferentiated fibroblasts into dopaminergic neurons. The iDA neurons that are generated synthesize dopamine and showed spontaneous electrical activity and are reversibly blocked by tetrodotoxin, which is consistent with the electrophysiological properties featured by brain dopaminergic neurons (De Gregorio et al., 2018).

### Spinal Motor Neurons

Genetic disorders like Amyotrophic Lateral Sclerosis (ALS) result in the loss of motor neurons (Robberecht and Philips, 2013). Regeneration of new motor neurons is important for potential therapy and disease models for ALS. Studies on mouse models have demonstrated that reprogramming of mouse embryonic fibroblasts into induced motor neurons (iMN) could be achieved by combined overexpression of common transcription factors [Ascl1, Neurog2, Myt1l, and Brn2 (Pou3f2)] with some specific TFs (Lhx3, Isl1, and Hb9) for spinal cord motor neurons (Lee et al., 2009; Son et al., 2011; Tang et al., 2017; Zhang et al., 2017). These iMNs could survive after being transplanted into the spinal cord and are capable of forming a neuromuscular junction with myotube cells *in vitro* (Son et al., 2011). To optimize the reprogramming condition, four TFs (Neurog2, Sox11, LHX3, and Isl1), when supplemented with forskolin, dorsomorphin, and FGF2 could directly reprogram human fibroblasts into motor neurons, which are HB9 and ChAT-positive, have action potentials and can form a neuromuscular junction with extremely high efficiency (>80%) (Liu et al., 2016).

### GABAergic Neurons (Interneurons)

The GABAergic neurons are inhibitory interneurons located in the cortex, which play crucial roles in regulating the excitation and inhibition of nervous system activation (Tremblay et al., 2016). The loss or malfunction of GABAergic neurons would also result in neurological diseases, such as epilepsies, cognitive disorders, autism, schizophrenia, and intellectual disabilities (Woo and Lu, 2006; Brooks-Kayal, 2010; Marin, 2012). Colasante et al. (2015) demonstrated the use of five TFs (Foxg1, Ascl1, Sox2, Dlx5, and Lhx6) for reprogramming human and mouse fibroblasts into induced GABA (iGABA) interneurons. The generated iGABA interneurons could survive and mature after being transplanted into the hippocampus (Colasante et al., 2015). The new iGABA interneurons can form functional synapses, and release GABA (Colasante et al., 2015). Importantly, the transplanted iGABA interneurons can integrate into host circuitry and play inhibitory functions (Colasante et al., 2015). A great part of the GABAergic neurons also showed Parvalbumin (PV) protein and gene expression. Soon after, another research group obtained induced PV (iPV) neurons by *Ascl1* from mouse fibroblasts (Shi et al., 2016). These reports showed that the controlled reprogramming process by some specific regional TFs would lead to lineage reprogramming of neuronal subtypes (Masserdotti et al., 2016).

## iPSCs Application for Neurodegenerative Diseases Modeling and Drug Discovery

**FIGURE 1:**
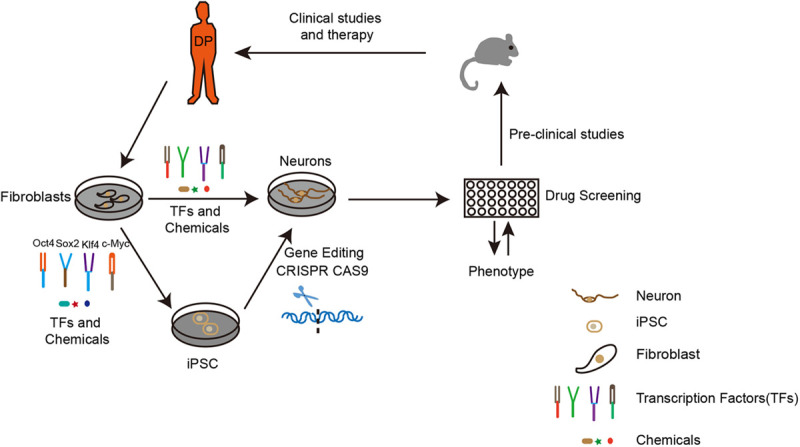
The route to apply iPSCs and iN technology for neurodegenerative disease modeling and drug discovery. To establish an *in vitro* disease model, the first step is to obtain fibroblasts from a diseased person. Upon overexpression of certain transcription factors, fibroblasts can be directly or indirectly reprogrammed to neurons. Depending on research purposes, the fibroblasts from original iPSCs can be modified by gene editing (CRISPR CAS9). The neurons generated would be applied for drug screening according to the disease phenotype. Subsequently, the best candidates could then be used for pre-clinical studies on drug toxicity, bio-availability, pharmacology, and metabolism in animals. Finally, the potential drug would be used for clinical studies and therapy. DP: disease person.

Neurodegenerative diseases including Alzheimer’s Disease (AD), Parkinson’s Disease (PD) and Amyotrophic Lateral Sclerosis (ALS) are aging-related disorders in which several genetic mutations have been identified before the onset of the diseases. However, even with a clearer understanding of the mechanisms of neurodegenerative diseases, the progression of designing therapy is going slow (Finkbeiner, 2010; Mason et al., 2014; Wyss-Coray, 2016). Based on these genetic mutations, different animal models have been established to study the underlying disease mechanisms and explore the potential drugs for treatment. Unfortunately, due to the variations among different species and the irreproducibility of human disease pathology, current animal models cannot ideally model neurodegenerative diseases as the data generated from these models cannot be successfully translated into clinical applications (Jucker, 2010; Imaizumi and Okano, 2014; Mitsumoto et al., 2014). In this scenario, iPSC technology brought new hope for neurodegenerative disease modeling and drug discovery *in vitro*. Nowadays, iPSC technology has been widely applied for disease modeling, mechanism study, and the screening of drugs for neurodegenerative diseases (Figure 1).

### Alzheimer’s Disease (AD)

Alzheimer’s Disease is the most common chronic progressive neurodegenerative disease. In recent decades, researchers have focused on the study of the pathogenesis of AD. Several genetic mutations have been identified in genes namely, APP, presenilin 1/2 (PS1/2), and APOE, to cause Familial Alzheimer’s disease (Armstrong, 2013; Karch et al., 2014; Shen, 2014; Moreno et al., 2018; Wang et al., 2018). However, the mechanisms of the neurons and synapse damage in AD remain unclear. The new iPSC technology from AD patients can provide sufficient quantity or quality neurons for the discovery of potential therapeutics (Byrne, 2014; Tcw, 2019).

In recent years, researchers have been successful in reprogramming fibroblasts carrying with different genetic mutations to iPSCs. Yagi et al. (2011) pioneered the use of iPSC technology to establish an *in vitro* model for AD, which was derived from iPSCs with PS1/2 mutation. The expression of Aβ42 was dramatically increased in neurons derived from iPSCs (Yagi et al., 2011). In addition to this, the APP gene mutation has also been investigated by Israel et al. (2012). They demonstrated that the levels of Aβ42 and tau are significantly increased in neurons derived from iPSCs (Israel et al., 2012; Ochalek et al., 2017). Subsequently, studies conducted by several groups of investigators used iPSCs to produce neurons derived to model AD where the properties of pathogenic Aβ42 and tau were reserved (Shi et al., 2012; Duan et al., 2014; Muratore et al., 2014; Sproul et al., 2014; Chang et al., 2015; Moore et al., 2015; Rowland et al., 2018; Tcw, 2019). In summary, the novel iPSCs *in vitro* model can be utilized as an excellent tool to study AD.

### Parkinson’s Disease (PD)

The loss of dopaminergic neurons in the neuropathology of PD, which causes motor problems, including bradykinesia, resting tremor, rigidity, flexed posture, “freezing,” and lose of postural reflexes (Seibler et al., 2011; Postuma et al., 2015). Similar to AD, the deficit of reliable *in vitro* models has limited the progression of drug discovery for PD. Several groups obtained iPSCs from patient somatic cells with different genetic mutations including LRRK2, SNCA, PARK2, or PINK1, which are related to familial PD, and the DA neurons derived from iPSCs has been used to investigate the molecular mechanisms (Ke et al., 2019). The dopaminergic neurons derived from LRRK2 iPSCs have some important PD features, including (α-Syn aggregates, overexpression of oxidative stress genes, lower number of neurites, and caspase-3 activation (Nguyen et al., 2011; Sanchez-Danes et al., 2012). Importantly, after correction of LRRK2 mutation in iPSCs, they can rescue the pathogenic phenotypes of neurite shortening and mitochondrial DNA damage (Reinhardt et al., 2013; Sanders et al., 2014). iPSCs derived from PD patients, who received triplication of the SNCA gene, have also been shown to have PD pathogenic neuron properties (Oliveira et al., 2015). PARK2 gene mutation has been shown to play a critical role in neuron morphology by iPSCs-derived neuron model (Imaizumi et al., 2012; Ren et al., 2015). In addition, (Seibler et al., 2011) also reported that iPSCs reprogrammed from PD patients’ fibroblasts with PINK1 mutations can generate DA neurons. The new DA neurons showed properties of upregulation of PGC-1α, which can be reversed after overexpression of wild-type PINK1 in new DA neurons. Together, all these studies demonstrated that iPSCs is a better *in vitro* model for PD with genetic mutations.

### Amyotrophic Lateral Sclerosis (ALS)

Induced Pluripotent Stem Cells technology also has been widely applied for ALS. The pathology of ALS includes the progressive loss of motor neurons in the brain and spinal cord. Several genes have been identified to be associated with ALS, such as SOD1, C9orf2, and TDP-43 (Rosen et al., 1993; Sreedharan et al., 2008; DeJesus-Hernandez et al., 2011). Among them, the SOD1 gene mutation is the most studied genetic alteration in ALS. Compare to wild type SOD1, motor neurons (MN) derived from SOD1 mutated patients’ iPSCs showed the features of decreased survival rate, smaller soma size, and shorter neurite (Chen et al., 2014; Kiskinis et al., 2014). In addition, MN derived from SOD1 mutated iPSCs showed impaired mitochondrial function and increased oxidative stress (Chen et al., 2014). Importantly, the correction of the SOD1 mutation could rescue these phenotypes in iPSCs (Chen et al., 2014; Kiskinis et al., 2014). iPSC-derived motor neurons retaining the patients’ full genetic information, therefore, scientists established a large number of *in vitro* cellular models for sporadic ALS. The sufficient utility of sporadic ALS models is useful for elucidating the pathological characteristics of specific cases and identifying novel candidate drugs (Fujimori et al., 2018). On the other hand, many investigators have studied the phenotypes of MN derived from C9orf72 mutant iPSCs (Donnelly et al., 2013; Sareen et al., 2013; Devlin et al., 2015; Dafinca et al., 2016). Abnormalities of electrophysiology, calcium homeostasis, ER stress, and mitochondrial membrane potential have been identified in MN from iPSCs carrying C9orf72 mutation (Devlin et al., 2015; Dafinca et al., 2016). In addition, the C9orf72 mutant has been demonstrated to cause oxidative and neurotoxicity in MN from iPSCs (Donnelly et al., 2013; Sareen et al., 2013; Birger et al., 2019).

## iN for Neurodegenerative Disease Modeling and Drug Discovery

With a specific combination of reprogramming factors, somatic cells can be directly converted into neurons bypassing the iPSC stage. Along with the advancement of direct reprogramming technology, the new generation of iN has also been applied for modeling neurogenerative diseases and drug discovery (Figure 1). Liu et al. (2016) have used direct reprogramming technology through using a combination of TFs and small molecules and efficiently reprogrammed ALS patients’ fibroblasts to motor neurons with FUS gene mutation. The new iMN from ALS patients was unable to form neuromuscular junctions with muscle cells. Moreover, after the chemical screening, they found the chemical kenpaullone can rescue the disease phenotype. Recently, (Chang et al., 2018) utilized the mesoporous silica nanoparticles (MSNs) as a non-viral delivery system for the transduction of the three key factors to achieve the conversion of mouse fibroblasts (MFs) into functional dopaminergic neuron-like cells. These recent studies are the beginning of developments that will enable us to apply iN for neurodegenerative disease modeling and drug discovery. Before applying this technique for large scale drug screening, the problems associated with efficiency and the homogeneity of direct reprogramming needs to be further improved.

## Comparison of iPSCs-Derived Neurons to iN

In contrast to the application of iPSC technology, the application of the iN approach is new and emerging in the field of neurodegenerative diseases. Like any technique, iN technology has some obvious advantages and disadvantages (Table 3). Because direct reprogramming does not involve the iPSC stage and the differentiation step, which saves a lot of time, iPSC technology may take several months, depending on the protocol. In addition, the technical challenges of iN are less compared to iPSCs culture technology. The most important difference between iPSCs and iN is epigenetic reset.

**TABLE 3:** The different features between iN and iPSCs-derived neurons.

**Features**	**iN**	**iPSCs-derived neurons**
Epigenetics reset	The generation of iN will not reset epigenetic information	Neuron derived from iPSCs will reset epigenetic information
Cell number and maintain	iN cell number is limited by original cell number and reprogramming efficiency, which are uneasy to maintain.	After acquisition of iPSCs, the production of neurons can be unlimited, which are easy to maintain.
Time for acquiring mature neurons	Directly reprogramming somatic cells to neurons only takes several weeks	Obtaining neurons derived from iPSCs will takes several months depending on protocol
Technical Challenges	Generation of iN using direct reprogramming technology is much simpler	iPSC technology of generation iN is complicated
Original cell types	Based on technology, the source for iN is limited (fibroblasts ect.)	The source for iPSCs is variable (fibroblasts, adipose stromal cells ect.)

As we know, a healthy and diseased person not only differs in genomic levels but also has different epigenetics. Epigenetic information is crucial for disease onset, especially for aging-related diseases. A recent study conducted by Tang et al. (2017) found iPSCs derived motor neurons did not show age-related differences, while iN, in contrast, age-equivalent induced motor neurons showed nuclear envelope defects. Mertens et al. (2015) provided interesting evidence for iN as it can reserve aging signatures of the original patient, which is not observed in iPSCs. Furthermore, they have also found downregulation of RanBP1 in aged fibroblasts and iN derived from aged fibroblasts, and when RanBP1 was knocked down, the transcriptional markers shifted from young to aged (Mertens et al., 2015). Therefore, iN is a more reliable model for neurodegenerative diseases and drug discovery, which could model natural disease progression, especially age-related information. On the other hand, iPSCs can maintain self-renewal but not iN, which is required for maintenance and stock. Due to the unlimited self-renewal of iPSCs, the neurons derived from iPSCs can be unlimited. Thus, without an iPSC stage, investigators might need to acquire a larger quantity of original cells from a patient to obtain enough iN. In addition, identification of the right combination of transcription factors, the inclusion of chemical compounds (small molecules), and the efficiency of reprogramming are also very important. However, to realize the application of iN in neurodegenerative diseases, the underlying mechanisms of direct reprogramming need to be further addressed.

## Conclusion and Perspective

In conclusion, this review has discussed recent iPSCs and iN technology and their application for neurodegenerative disease modeling. Compared them to traditional disease models, both iPSCs and iN are more accurate models for studying diseases and drug discovery. For iPSCs and iN disease models, there are still some challenges that need to be further investigated to optimize reprogramming conditions, especially the efficiency of direct reprogramming and lineage-specific reprogramming. For the modeling of neurodegenerative diseases, iN could be a better model for disease and the development of drugs, without epigenetic reset. In the coming years, we expect there to be extensive improvements in reprogramming technology for the application of iPSCs and iN for disease modeling and drug discovery.

## Author Contributions

YZ, XX, and JY performed the literature research and wrote the manuscript. C-LZ and QZ proposed the framework. JH and KA critically revised the manuscript. QZ and JY provided the funding support. All authors read and approved the manuscript.

## Conflict of Interest

The authors declare that the research was conducted in the absence of any commercial or financial relationships that could be construed as a potential conflict of interest.

## Abbreviations

ABM: Ascl1, Brn2, and Myt1l
AD: Alzheimer’s Disease
ALS: Amyotrophic Lateral Sclerosis
ANL: Ascl1, Nurr1, and Lmx1a
APOE: apolipoprotein E
APP: amyloid beta precursor protein
ASCL1: achaete-scute family BHLH transcription factor 1
Brn2: POU domain, class 3, transcription factor 2
Dlx2: distal-less homeobox 2
Dlx5: distal-less homeobox 5
FGF2: fibroblasts growth factor 2
FoxA1/2: forkhead box A1/2
Foxg1: forkhead box G1
Hb9: motor neurons and pancreas homeobox 1
iDA: induced dopaminergic
iMN: induced motor neurons
iN: induced neurons
iPSCs: induced pluripotent stem cells
Isl1: ISL LIM homeobox 1
Klf4: Kruppel-like factor 4
Lhx3: LIM homeobox protein 3
Lhx6: LIM homeobox protein 6
Lmx1a/b: LIM homeobox transcription factor 1 a/b
LRRK2: leucine rich repeat kinase 2
Myt1l: myelin transcription factor 1 like
Ngn2: neurogenin 2
Nurr1: (Nr4a2), nuclear receptor subfamily 4, group A, member 2
Oct4: octamer-binding transcription factor 4
OHDA: hydroxydopamine
OSKM: Oct4, Sox2, Klf4, and c-Myc, otx2, orthodenticle homeobox 2
PARK2: parkin RBR E3 ubiquitin protein ligase
PD: Parkinson’s Disease
PGC-1 α: PPARG coactivator 1 alpha
PINK1: PTEN induced putative kinase 1
Pitx3: paired-like homeodomain transcription factor 3
PS1/2: presenilin 1/2
PV: Parvalbumin
RanBP1: RAN binding protein 1
SNCA: synuclein alpha
SOD1: superoxide dismutase 1
Sox11: SRY (sex determining region Y)-box 11
Sox2: SRY (sex determining region Y)-box 2
TDP-43: TAR DNA binding protein
TFs: transcription factors
Zfp521: Zinc Finger Protein 521.
